# Efficient Multi-Sites Genome Editing and Plant Regeneration *via* Somatic Embryogenesis in *Picea glauca*

**DOI:** 10.3389/fpls.2021.751891

**Published:** 2021-10-14

**Authors:** Ying Cui, Jian Zhao, Ying Gao, Ruirui Zhao, Jinfeng Zhang, Lisheng Kong

**Affiliations:** ^1^Beijing Advanced Innovation Center for Tree Breeding by Molecular Design, College of Biological Science and Biotechnology, Beijing Forestry University, Beijing, China; ^2^Department of Biology, Centre for Forest Biology, University of Victoria, Victoria, BC, Canada

**Keywords:** conifer, *Picea glauca*, somatic embryogenesis, genome editing, CRISPR/Cas9, *DXS1*, plant regeneration, gymnosperm

## Abstract

Conifers are the world's major source of timber and pulpwood and have great economic and ecological value. Currently, little research on the application of CRISPR/Cas9, the commonly used genome-editing tool in angiosperms, has been reported in coniferous species. An efficient CRISPR/Cas9 system based on somatic embryogenesis (SEis) suitable for conifers could benefit both fundamental and applied research in these species. In this study, the *SpCas9* gene was optimized based on codon bias in white spruce, and a spruce *U6* promoter was cloned and function-validated for use in a conifer specific CRISPR/Cas9 toolbox, i.e., *PgCas9/PaU6*. With this toolbox, a genome-editing vector was constructed to target the *DXS1* gene of white spruce. By *Agrobacterium*-mediated transformation, the genome-editing vector was then transferred into embryogenic tissue of white spruce. Three resistant embryogenic tissues were obtained and used for regenerating plants *via* SEis. Albino somatic embryo (SE) plants with mutations in *DXS1* were obtained in all of the three events, and the ratios of the homozygous and biallelic mutants in the 18 albino mutants detected were 22.2% in both cases. Green plants with mutations in *DXS1* were also produced, and the ratios of the *DXS1* mutants to the total green plants were 7.9, 28, and 13.5%, respectively, among the three events. Since 22.7% of the total 44 mutants were edited at both of the target sites 1 and 2, the CRISPR/Cas9 toolbox in this research could be used for multi-sites genome editing. More than 2,000 SE plants were regenerated *in vitro* after genome editing, and part of them showed differences in plant development. Both chimerism and mosaicism were found in the SE plants of white spruce after genome editing with the CRISPR/Cas9 toolbox. The conifer-specific CRISPR/Cas9 system developed in this research could be valuable in gene function research and trait improvement.

## Introduction

Conifers are gymnosperms that are distributed in all continents except Antarctica. They are the dominant trees in many areas, especially where limited water and cold temperature restrict plant growth (Neale and Wheeler, 2019). Besides their contributions to forest ecosystems, conifers are also the major source of timber and pulpwood. In 2019, about 64.8% sawlogs and veneer logs, as well as 51.4% pulpwood, were produced from conifers, and the plantation area of conifers accounted for 31% of the total global forest plantation area (FAOSTAT, http://www.fao.org). Due to the economic and ecological importance, fundamental studies and trait improvements are needed for coniferous species. At present, trait improvement of conifers is mainly achieved through breeding with the selected elite plants and the offspring screening assisted by molecular methods. This process usually takes a long period of time. New methods to introduce target traits or modify the original traits are thus urgently needed to speed up breeding in conifers.

The sizes of the conifer genomes are enormous, ranging from 20 to 40 Gb (De La Torre et al., 2015; Cañas et al., 2019). Several conifer genomes have been published, such as white spruce (*Picea glauca*) (Birol et al., 2013), Norway spruce (*Picea abies*) (Nystedt et al., 2013), loblolly pine (*Pinus taeda*) (Zimin et al., 2014), sugar pine (*Pinus lambertiana* Dougl.) (Stevens et al., 2016) and larch (*Larix kaempferi*) (https://www.ncbi.nlm.nih.gov/assembly/GCA_013171265.2). There are also lots of transcriptome data in the public database, such as CONGENIE (https://congenie.org). But gene functional research in conifers is still lagging compared to that in the woody species of angiosperms, such as poplar. In addition to the long growth cycle of conifers, the lack of an efficient transgenic system and a genome-wide mutant library also restricts studies on gene functions and trait improvement in conifers. Additionally, research of conifers based on angiosperm model systems, such as *Arabidopsis*, is often less informative, since gymnosperms and angiosperms began to differ 300 million years ago (De La Torre et al., 2020). Thus, molecular tools suitable for coniferous species are strongly required.

Genome editing is a powerful technology for functional genomic research and trait improvement. To date, several tools have been successfully used in genome editing, such as zinc finger nucleases (ZFNs), transcription activator-like effector nucleases (TALENs), and clustered regularly interspaced short palindromic repeats/CRISPR-associated proteins (CRISPR/Cas); among of which, CRISPR/Cas is the most widely applied tool due to its high mutagenesis efficiency and easy application (Mao et al., 2019). The CRISPR/Cas system can be divided into several types according to the effector proteins, and CRISPR/Cas9 belongs to the type II system, which is characterized by a single large protein in the expression and interference modules (Makarova et al., 2015). Among different systems of CRISPR/Cas, CRISPR/Cas9 was used firstly for plant genome editing (Feng et al., 2013). The CRISPR/Cas9 system recognizes the target site with an adjacent motif (PAM), which is usually “NGG,” and causes a double-strand break (DSB) at about 3 bp upstream of the PAM sequence. When the DSB is repaired by non-homologous end joining (NHEJ), mutations may happen at this site (Mali et al., 2013). CRISPR/Cas9 has been applied successfully in a large number of angiosperms, including various woody plants, such as poplar (Zhou et al., 2015; Wang et al., 2020), citrus (Jia and Wang, 2014; Peng et al., 2017; Dutt et al., 2020), grape (Ren et al., 2016; Wang X. et al., 2018), apple (Nishitani et al., 2016), kiwifruit (Varkonyi-Gasic et al., 2019), pomegranate (Chang et al., 2019), and so on. However, in gymnosperms, there were only limited reports about genome editing with CRISPR/Cas9. For example, a PAM-less Cas9 variant SpRY was confirmed to have the ability of genome editing with the protoplast of Dahurian larch (*Larix gmelinii*). However, no genome-edited plants were regenerated in this research (Ren Q. et al., 2021). Most recently, CRISPR/Cas9-mediated-targeted mutagenesis has been reported in both *Pinus radiata* (Poovaiah et al., 2021) and *Cryptomeria japonica* (Nanasato et al., 2021), which indicated the feasibility of applying CRISPR/Cas9 in conifers. These reports were very exciting, but a high frequency of chimera was found in both of the studies. It will be interesting to edit genomes of some other coniferous species with different CRISPR/Cas9 toolboxes for further study on CRISPR/Cas9-mediated mutagenesis in conifers.

Although the CRISPR/Cas system is very efficient, its application in higher plants was limited by plant regeneration (Mao et al., 2019). Somatic embryogenesis (SEis) is one of the most efficient methods of plant propagation or regeneration, especially for conifers (Maruyama and Hosoi, 2019). The SEis method possesses obvious advantages over the other methods. One advantage is that thousands of somatic embryos (SEs) with both shoot and root meristems could be produced from a small amount of embryogenic tissues (Isah, 2016). In addition, the selected embryogenic cell lines could be conserved for a long period of time using the technology of cryopreservation (Cyr, 1999). Thus, the advantage of SE is the attribute efficient propagation of genome-edited plants in coniferous species, which usually have long sexual reproduction cycles.

Because the albino phenotype is easily visualized, it is commonly used as a marker for targeted genome editing in plants (Bewg et al., 2018). The *CLA1* (for “*cloroplastosalterados*”) gene encodes 1-deoxyxylulose 5-phosphate synthase (DXS), which is the first enzyme in the 2-C-methyl-D-erythritol-4-phosphate (MEP) pathway and is an essential gene for the synthesis of chlorophyll and carotenoids (Mandel et al., 1996; Estévez et al., 2000). In cotton, knocking out the *CLA1* gene using CRISPR/Cas9 resulted in albino mutants (Wang P. et al., 2018). In Norway spruce, three *DXS* genes were reported, and one of which, *DXS1*, was predicted to be involved in the synthesis of chlorophyll and carotene according to its expression profile (Phillips et al., 2007).

In this study, the *SpCas9* gene was optimized according to codon bias in white spruce, and a spruce *U6* promoter was cloned and verified. These two elements were used to construct a conifer specific CRISPR/Cas9 toolbox. With this toolbox, we successfully knocked out the *DXS1* gene in white spruce after *Agrobacterium*-mediated transformation based on a stable SEis system. Albino mutants with either homozygous mutations or biallelic mutations at the target site were obtained at the T_0_ generation. Our research confirmed that the application of CRISPR/Cas9 in white spruce was feasible, but chimerism and mosaicism existed widely during genome editing with CRISPR/Cas9. Our results could benefit further applications of CRISPR/Cas9 in coniferous species.

## Materials and Methods

### Construction of CRISPR/Cas9 Toolbox Suitable for Conifers

In order to construct CRISPR/Cas9 toolbox suitable for conifer transformation, *Streptococcus pyogenes* Cas9 (*SpCas9*) fused with a nuclear localization signal (NLS) at each side of the 5′ and 3′ end was optimized according to codon bias in white spruce with GenScriptOptimumGene^TM^ tool (Genscript, Piscataway, NJ08854, USA) and designated as *PgCas9*. The doubled *CaMV35S* promoter and *Nos* terminator were added, respectively, at the 5′ end and 3′ end of the *PgCas9* to construct the *PgCas9* expression cassette.

In order to clone *U6* promoters, which could express in conifers, homologous sequences of the *AtU6-26*gene (GenBank: X52528.1) were searched in the genome sequences of white spruce and Norway spruce, which were published in the CONGENIE database (https://congenie.org/) with BLAST tool. Then, the promoter sequences of these homologous sequences were analyzed. The candidate *U6* promoters were amplified from Norway spruce embryogenic cell line 1 (NP1) and white spruce embryogenic cell line 3 (WSP3), both of which were induced and preserved in our lab. After sequencing analysis, the cloned *U6* promoter was constructed into the binary vector pDX2181 (provided by Prof. Yongjun Lin, National Key Laboratory of Crop Genetic Improvement, Huazhong Agricultural University) (Wang et al., 2015), in which there was *GUS* gene terminated with *Nos* terminator used for the promoter functional analysis. The constructed vector was subsequently introduced into the embryogenic tissue of white spruce (WSP3) and Prince Rupprecht's larch (*Larix principis-rupprechtii* Mayr.) (cell line of 25a, induced and preserved in our lab) by *Agrobacterium*-mediated transformation. After 5–7 days of selection culture, histochemical staining of GUS activity in the infected embryogenic tissue was carried out, following the manufacturer's introduction of GUS staining solution (G3061, Beijing Solarbio Science and Technology CO., Ltd., Beijing, China). Briefly, about 50 mg of infected embryogenic tissues were incubated in 500 μl of GUS-staining solutions and incubated at 37°C for 10 h; after which, the samples were incubated in 70% ethanol for 1 h and were photographed under a dissecting microscope (Leica S9i).

The functioning spruce *U6* promoter was then constructed at the upstream of the gRNA backbone, which had been described by Xie et al. (2015). The final sequence containing *PgCas9*-expressing cassette and *U6* promoter: gRNA backbone was synthesized by Genscript Biotechnology Co., Ltd (Nanjing, China) and ligated into the modified pCAMBIA1300 vector, in which the *Bsa*I site was removed with QuickMutation^TM^ (D0206, Beyotime Biotechnology, Shanghai, China), following the manufacturer's introduction.

### Construction of the Genome-Editing Vector Targeting at the *DXS1* Gene

The *DXS1* gene in white spruce was obtained by searching the homologous sequence of the published *DXS1* gene (Phillips et al., 2007) in the CONGENIE database, and the primers, *DXS*-4F (5′-CTGCAGGCTTATCCTCACAAG-3′) and *DXS*-5R (5′-CAGCTTACTCAGTGCACTGC-3′), were designed according to the sequence of white spruce *DXS1* gene. After that, genomic DNA of white spruce embryogenic cell line WSP3 was extracted with the Super Plant Genomic DNA kit (Polysaccharides and Polyphenolics-rich) (DP360, TIANGEN Biotech CO., Ltd., Beijing, China) according to the manufacturer's instruction and was used as the template to amplify the *DXS1* gene. PCR was carried out with 50 ng of white spruce genomic DNA, 5 μl of 10 × KOD buffer, 5 μl of dNTP (8 mM), 2 μl of MgSO_4_ (25 mM), 1.5 μl of each *DXS*-4F (10 μM), and *DXS*-5R (10 μM), 1 μl of KOD-plus DNA polymerase (KOD-401, TOYOBO Co., Ltd., Osaka, Japan), in a total volume of 50 μl. The PCR conditions were 94°C for 2 min, then 30 cycles of 94°C for 15 s, 58°C for 30 s, 68°C for 30 s, and finally, 68°C for 5 min. PCR products were sequenced, and the sequence was used for designing gRNA1 and gRNA2 with the online tool CRISPOR (Concordet and Haeussler, 2018). After that, the polycistronic tRNA-gRNA (PTG) structure for gRNA1 and gRNA2 was assembled according to the golden gate assembly method described by Xie et al. (2015) and then ligated into the CRISPR/Cas9 toolbox. The detailed procedure of constructing the genome-editing vector targeting at the *DXS1* gene is shown in Supplementary Method 1. The final genome editing vector was introduced into *Agrobacterium* (*EHA*105) by electroporation for subsequent use in plant transformation.

### Plant Materials for Transformation

Embryogenic tissue was induced from zygotic embryos dissected from mature seeds of white spruce. Embryogenic tissue of WSP3 was selected on its *in vitro* culture performance, i.e., the stable tissue maintenance and high yield in SE and plant production, and used for *Agrobacterium*-mediated transformation in this research. All the reagents used in SEis and *Agrobacterium*-mediated transformation were purchased from Sigma-Aldrich® (St. Louis, MO, USA). Half-strength modified Litvay's medium (1/2 mLV, Litvay et al., 1985; Kong and von Aderkas, 2007) was used as the basal medium. The final pH was 5.8 for all medium types except the co-culture medium. Embryogenic tissue was induced on the induction medium containing 2 mg/L 2,4-dichlorophenoxyacetic acid (2,4-D), 1 mg/L 6-benzylamino adenine (6-BA), 0.5 g/L hydrolyzed casein, 0.5 g/L glutamine, 10 g/L sucrose, and 3 g/L gellan gum (phytagel^TM^). During maintenance, embryogenic tissue was transferred onto a solid maintenance medium, which was an induction medium with 2,4-D and 6-BA of half strength. After two sub-cultures, 0.8 g of WSP3 embryogenic tissue was inoculated into one flask, containing 50 ml of the liquid maintenance medium without a gelling agent, and cultured on a shaker at 100 rpm, 23°C, in darkness, and sub-cultured one time per week. The suspended embryogenic tissue was then transferred into the liquid pretreatment medium supplemented with 30 μM abscisic acid (ABA), 0.4 g/L hydrolyzed casein, 0.5 g/L glutamine, and 10 g/L sucrose, and cultured under the same culture conditions for one week before the tissue was ready for a transformation.

### *Agrobacterium*-Mediated Transformation and Plant Regeneration

*Agrobacterium EHA*105 containing the desired vector was cultured on Luria-Bertani medium (Bertani, 1951), containing 5 g/L yeast extract, 10 g/L Tryptone, 10 g/L NaCl, 16 g/L Agar powder, and 50 mg/L kanamycin at 28°C for 2 days, and then cultured in 50 ml of co-culture medium (supplemented with 1 mg/L 2,4-D, 0.5 mg/L 6-BA, 0.5 g/L hydrolyzed casein, 0.5 g/L glutamine, 10 g/L sucrose, and 50 μM acetosyringone, pH 5.2) on a shaker at 180 rpm, 28°C for 2 h. The OD_600_ of the *Agrobacterium* suspension was then adjusted to 0.5 with co-culture medium, after which, the *Agrobacterium* suspension was used to infect the pre-treated embryogenic tissue of WSP3 in white spruce or 25a in Prince Rupprecht's larch for 15 min. Subsequently, the infected embryogenic tissue was transferred onto a sterile filter paper, which had been moistened with 1 ml of co-culture medium, and cultured in the dark at 25°C for 3 days. After that, *Agrobacterium* was removed from the infected embryogenic tissue by washing embryogenic tissue with the liquid maintenance medium one time, and then soaking in the liquid maintenance medium containing 400 mg/L cefotaxime sodium for 30 min. The rinsed embryogenic tissue was then cultured on a maintenance medium containing 400 mg/L cefotaxime sodium for 7 days at 23°C in the dark. Subsequently, the embryogenic tissue was transferred onto the selection medium, which was the maintenance medium supplemented with 10 mg/L hygromycin and 400 mg/L cefotaxime sodium, and cultured at 23°C in the dark for 4–6 weeks with subcultures biweekly. The hygromycin-resistant tissue was further proliferated on the selection medium for 14 days, and then suspended in the liquid maintenance medium for 7 days on a shaker at 100 rpm, 23°C. The suspended resistant embryogenic tissue was cultured in the liquid pretreatment medium for 7 days and transferred to the maturation medium, containing 45 μM ABA, 0.2-g/L hydrolyzed casein, 0.4 g/L glutamine, 30 g/L sucrose, 10 g/L maltose, and 6 g/L phytagel. After a 30-day maturation, the cotyledonary SEs were collected and placed in a 4°C incubator at least for 10 days, and then transferred onto the germination medium, containing 0.5 g/L NH_4_NO_3_, 0.5 g/L glutamine, 10 g/L sucrose, 2 g/L activated carbon, and 3.2 g/L gellan gum. Germination of SEs was performed under a low-intensity light for the first 7 days and then under a high-intensity light until the SEs converted into SE plants.

To calculate tissue proliferation rates, 0.2 g embryogenic tissue of the wild type and the resistant ones were put on the maintenance medium proximately and cultured for 7 days, respectively. The proliferation rate was then obtained by dividing the weight of embryogenic tissue after proliferation by its original weight. SE maturation efficiency was evaluated 30 days after maturation by calculating the number of SEs with well-developed cotyledons generated from each gram of embryogenic tissue inoculated.

### Detection of Mutations in the *DXS1* Gene

Genomic DNA of the whole SE plants, 20 days after germination, was extracted with a DNALyse Amplification kit (CW0556S, ComWin Biotech Co., Ltd., Beijing, China). PCR amplification of *hpt* was then performed with the primers *hpt*-F (5′-ACACTACATGGCGTGATTTCAT-3′) and *hpt*-R (5′-TCCACTATCGGCGAGTACTTC T-3′) according to the instruction of the DNALyse Amplification kit. After that, two rounds of PCR were performed to amplify the *DXS1* gene. The primers for the first and second rounds of PCR were *DXS*-4F-2 (5′-GCTGCGACTTAACATTCTAC-3′) and *DXS*-5R, *DXS*-4F, and *DXS*-5R, respectively. The PCR system for the two rounds of PCR was the same as that described previously. The sequences of the second round of PCR products were then obtained by Sanger sequencing and analyzed with the tool DSDecode (Liu et al., 2015). Mutations in the *DXS1* were also analyzed by the Hi-TOM platform (Liu et al., 2019) by preparing PCR products according to the instruction of the platform with the primer *DXS*-T1F (5′-GGAGTGAGTACGGTGTGCCAGACAAATGGCCTCTCAGG-3′) and *DXS*-T2R (5′-GAGTTGGATGCTGGATGGCATAGCTTCAAAGGCTTGCC-3′). The mutation frequency and the type in the plants were then carefully analyzed. The phenotype of the SE plants was also examined, and the ratio of different types of SE plants was calculated.

### Statistical Analysis

Statistical analysis was completed with at least three independent replicates (*N* ≥ 3), subjected to one-way analysis of variance (ANOVA). Differences with *p* < 0.05 were considered significant.

## Results

### A Spruce *U6* Promoter Was Cloned, and a CRISPR/Cas9 Toolbox Suitable for Conifers Was Constructed

The *Arabidopsis U6* (*AtU6*) gene promoter contains two conserved upstream elements, the USE with sequence 5′-RTCCCACATCG-3′ and a TATA element (Waibel and Filipowicz, 1990). According to the *AtU6-26* gene sequence, six fragments with identity (>96%) were obtained from the genomes of Norway spruce and white spruce, respectively. The 500 bp upstream sequences of these fragments were analyzed. Two upstream sequences from Norway spruce and two upstream sequences from white spruce showed a conserved sequence 5′-TCCCACATGC-3′, which was similar to the USE sequence in the *AtU6* promoter. The four upstream sequences were PCR amplified by the designed primers according to the spruce reference genome. However, only one upstream sequence from Norway spruce was amplified successfully. This upstream sequence was designated as *PaU6* promoter (Supplementary Table 1) and used to drive *GUS* expression to verify its function. As GUS activity could be detected in embryogenic tissue of both white spruce and Prince Rupprecht's larch after *Agrobacterium*-mediated transformation (Supplementary Figure 1), the *PaU6* promoter was proved functional. Hence, the *PaU6* promoter was used to construct the conifer genome-editing binary vector.

**Figure 1:**
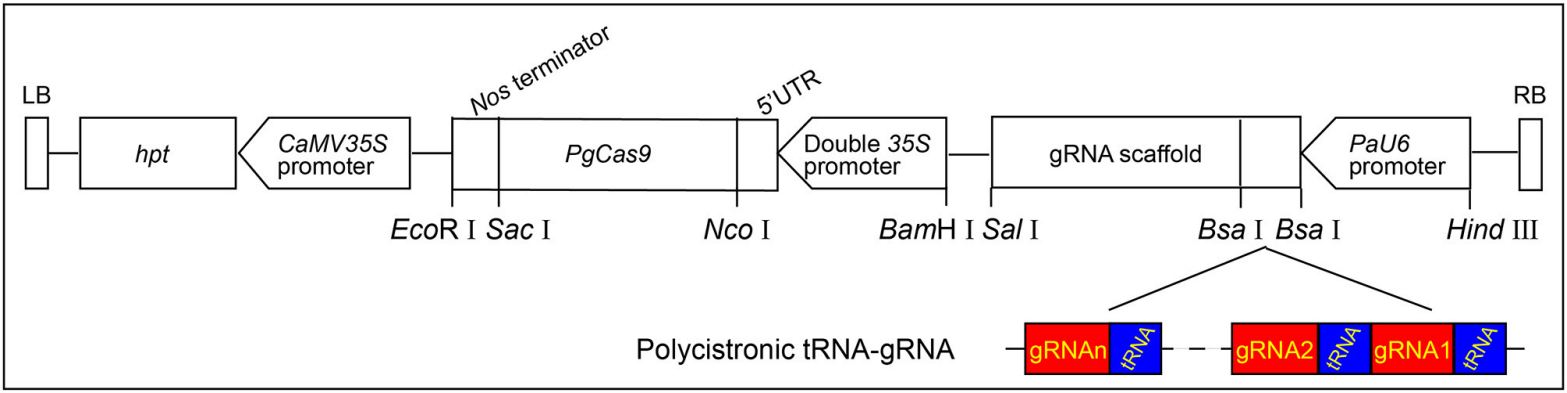
The T-DNA structure of *PgCas9*/*PaU6*. The *hygromycin phosphate transferase* (*hpt*) gene, which was driven and terminated by the *CaMV35S* promoter and the *CaMV35S* polyA, respectively, was used as the selective marker gene. The *PgCas9* with a nuclear localization signal (NLS) at each side of 5′ and 3′ ends was driven by the doubled *35S* promoter and terminated by the *Nos* terminator. The *PaU6* promoter was used to drive the expression of polycistronic tRNA-gRNA (PTG), and two *Bsa*I sites were designed between the *PaU6* promoter and the gRNA backbone for assembling the PTG into the binary vector. *Eco*RI, *Sac*I, *Nco*I, *Bam*HI, *Sal*I, and *Hind*III were restriction enzymes. The left and right borders of T-DNA were designated as LB and RB, respectively.

As the optimal codons in conifers usually possess A or T at the third codon position, which was very different from those of other plant species (Serres-Giardi et al., 2012; De La Torre et al., 2015), the *SpCas9* gene was optimized according to codon bias in white spruce and designated as *PgCas9*. After optimization, the GC content of *PgCas9* was 36.94%, and the Codon Adaption Index (CAI) in white spruce was 0.93 (analyzed with GenScriptOptimumGene^TM^ tool), which meant that *PgCas9* could be translated efficiently in white spruce. The final binary vector containing the *PgCas9*-expressing cassette and the *PaU6* promoter: gRNA backbone sequence was designated as *PgCas9/PaU6*. The detailed information is provided in Figure 1.

### Transgenic Embryogenic Tissues and SE Plants Showed Varied Phenotypes

**Figure 2:**
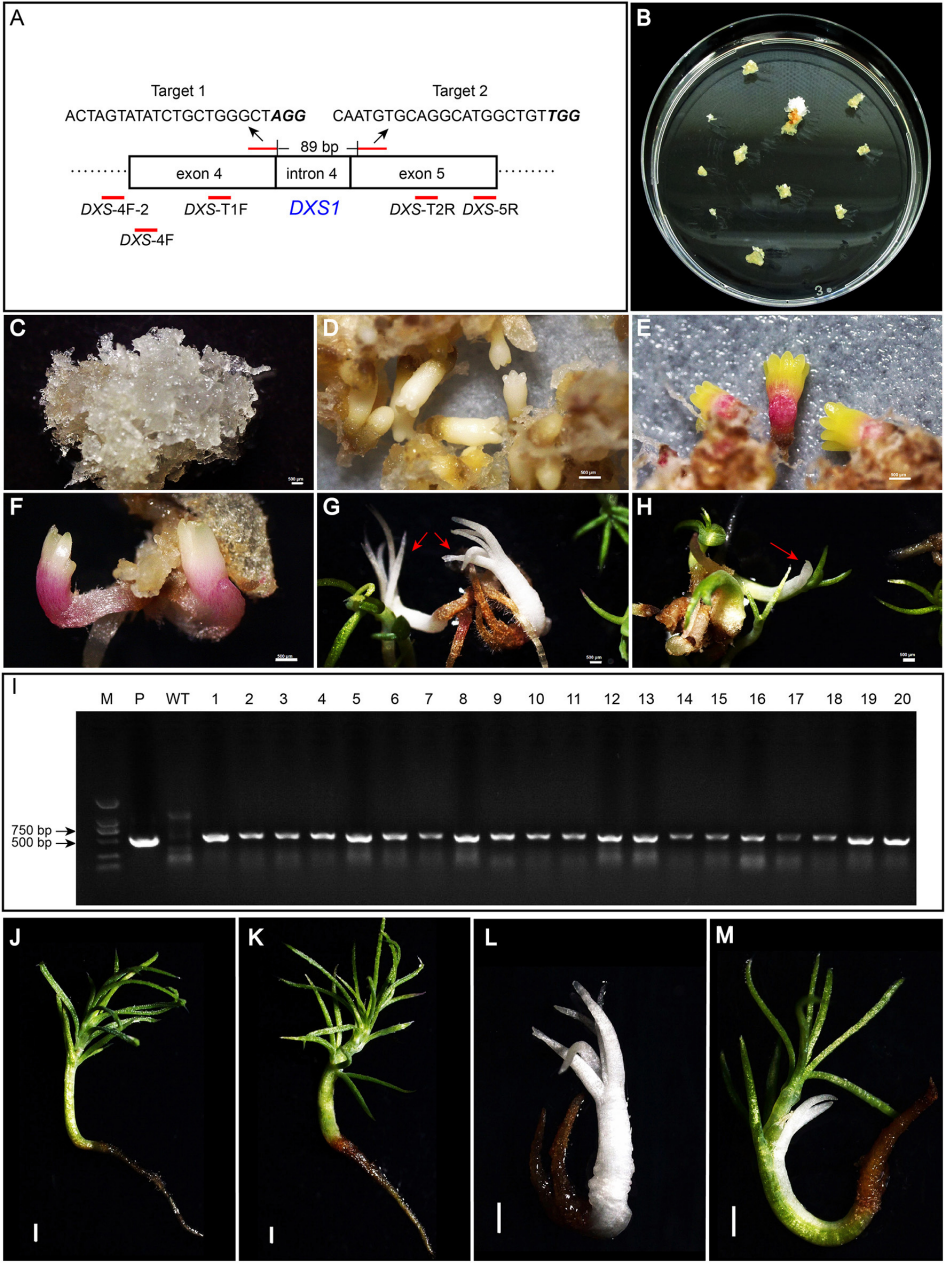
Target sites in the *DXS1* gene and regeneration of transgenic somatic embryo (SE) plants. The sequences of two target sites in the *DXS1* gene and the primers used for the detection of mutations were shown in **(A)**. Letters in italic bold type were PAM sites. The primers were shown under the *DXS1* gene with their names and positions indicated. Hygromycin-resistant embryogenic tissue was obtained about 4 weeks after transformation **(B)**. Resistant embryogenic tissues proliferated quickly on the selection medium **(C)**. Cotyledonary SEs developed after 4 weeks of maturation **(D)** and further converted into SE plants on the germination medium **(E–H)**. **(E,F)** showed the germination of green and albino SE plants, respectively. **(G,H)** showed the growth of albino and mosaic SE plants after 20 days on the germination medium, which was indicated with red arrows. **(I)** showed the results of PCR amplification of *hpt* gene in the transgenic SEs (1–20), wild-type SEs (WT), and the positive control *PgCas9/PaU6* (*DXS1*) (P). SE plants of wild type and transgenic types, including green, albino, and mosaic ones after 65 days on the germination medium, were shown in **(J–M)**, respectively. All bars equal to 500 μm in **(C–H)**, while bars in **(J–M)** equal to 1,000 μm.

When searching for the white spruce *DXS1* gene according to the sequence of the published Norway spruce *DXS1* gene, only a single DNA fragment was found, indicating that the white spruce *DXS1* gene was a single copy gene. By PCR amplification and Sanger sequencing, the accurate sequence between the fourth and fifth exon of the *DXS1* gene was obtained in white spruce embryogenic cell line WSP3 (Supplementary Table 1). By searching the target sites in CRISPOR, two target sites with a predicted high-efficiency score were selected to construct the genome-editing vector, targeting at the *DXS1* gene. Detailed information about the two target sites is provided in Figure 2A.

*Agrobacterium*-mediated transformations were then carried out two times, and a total of three resistant embryogenic tissues (designated as R1, R2, and R3) were obtained (Figure 2B). Tissue proliferation rates during solid maintenance cultures differed significantly between the three transgenic events and the wild type (Table 1). All of the embryogenic tissues could proliferate quickly (Figure 2C). Compared with the wild type, SE maturation demonstrated significant differences in R2 and R3, except R1 (Table 1). Similar to the wild type, the resistant embryogenic tissues produced mature SEs with well-developed cotyledons (Figure 2D). During germination, most mature SEs turned green, but some of them developed SEs of the albino phenotype (Figures 2E,F). The albino SEs remained white in color during germination culture (Figure 2G). Besides green and albino SE plants, mosaic SE plants with both albino and green colors were also observed (Figure 2H). The ratios of albino and mosaic plants to the total plants can be found in Table 2. Either R2 or R3 produced albino SE plants over 2% of the total.

**Table 1:** The proliferation of embryogenic tissue and maturation of SEs before and after genome editing.

	**Wild type**	**R1**	**R2**	**R3**
Tissue proliferation (fold/wk)	5.07, 0.86	3.16, 0.52^*^	2.71, 0.27^*^	2.76, 0.50^*^
Mature SEs (SEs/g FW)	501, 64	483, 53	223, 55^*^	184, 44^*^

*Means ± SD, n = 3 for tissue proliferation; n = 9 for embryo maturation*.

* *Stands for the significant difference at p < 0.05 when compared with the wild type*.

**Table 2:** The frequency of the visible transgenic SE plants to the total regenerated plants.

**Transgenic events**	**Total SE plants**	**Number of albino plants**	**Number of mosaic plants**
R1	1,139	2	1
R2	532	12	1
R3	503	11	1

A total of 50–60 SE plants were then randomly selected from each of the three transgenic events. These plants were confirmed to be transgenic as the *hpt* gene could be amplified from all of them except the wild type (Figure 2I). When the SE plants were further cultured on the germination medium, most of the transgenic, green plants could develop healthy shoots and roots, comparable to those of wild-type WSP3 SE plants, whereas the albino transgenic plants failed in shoot development and the mosaic transgenic plants grew slower obviously (Figures 2J–M). Since albino SE plants of white spruce were very rare in the control, we speculated that the albino phenotype was caused by mutations in the *DXS1* gene.

### Targeted Mutations Happened in the *DXS1* Gene

**Figure 3:**
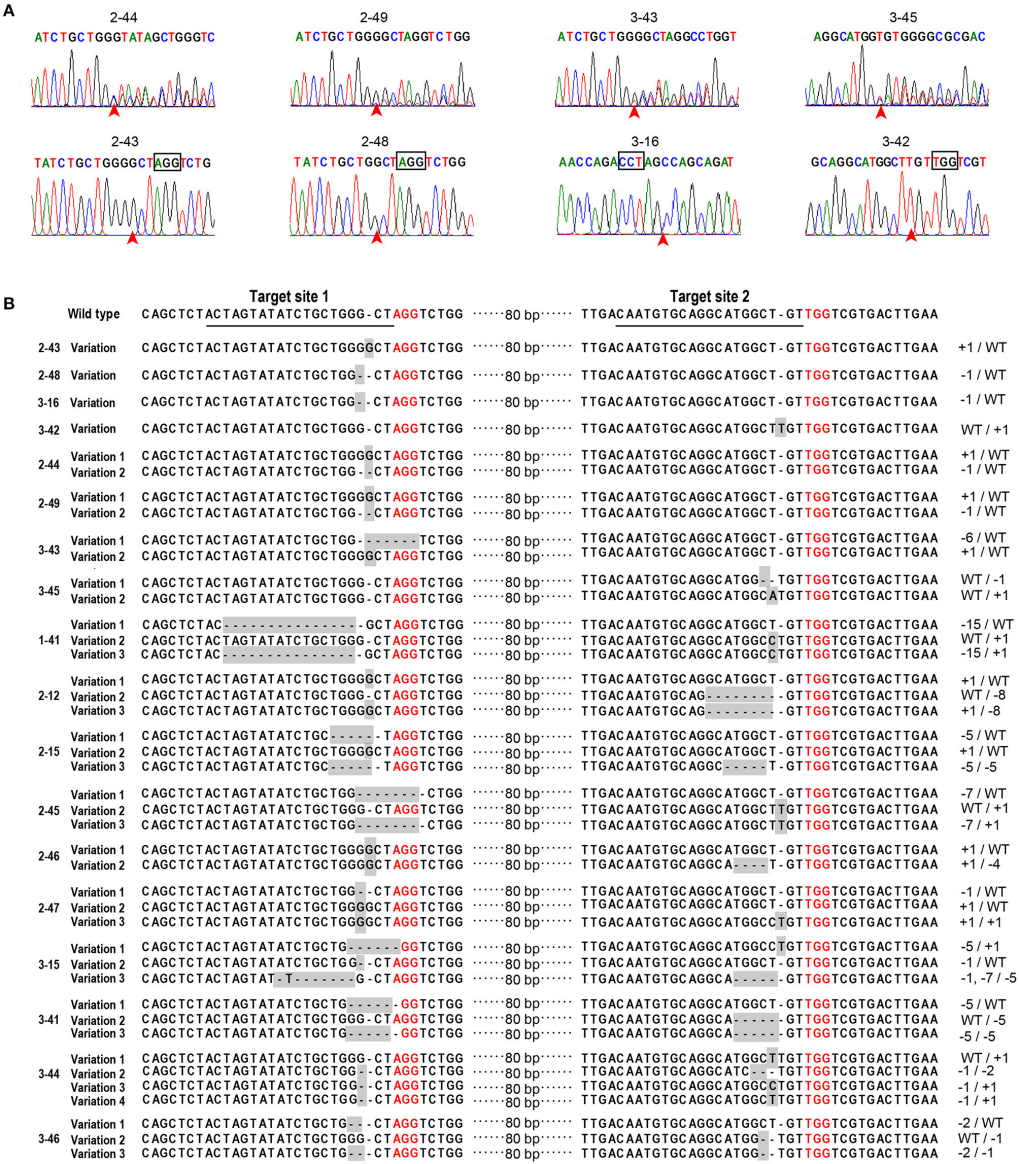
Sequences at the target sites in albino SE plants. Sequencing chromatograms of biallelic (2–44, 2–49, 3–43, and 3–45) and homozygous (2–43, 2–48, 3–16, and 3–42) mutations were shown in **(A)**. The red arrows indicated the sites of mutations. The edited sequences in the 18 albino SE plants were listed in **(B)**. The sequences at the target site 1 and the target site 2 in wild-type SE plants of WSP3 were underlined. PAM sites were indicated with red color. The line number of the individual transgenic line was indicated on the left side of the sequence. The nucleotide insertion and deletion were indicated by +n bp and -n bp, respectively, on the right side of the sequence.

A total of 165 green and 18 albino SE plants from the three transgenic events were used to evaluate the genome-editing efficiency and the character of the mutation. The sequence of PCR product amplified with the primer *DXS*-4F and *DXS*-5F was analyzed with the tool DSDecode, and the result indicated that mutations in the *DXS1* gene existed in a total of 44 SE plants. Most sequencing chromatograms of the mutations were characterized by double peaks (Figure 3A), and the decoding results indicated that they were biallelic mutations (the same target site in both homologous chromosomes was mutated but with different mutations) or heterozygous mutations (the target site in only one homologous chromosome was mutated). Some other mutations demonstrated a simple sequencing chromatogram but were decoded to be homozygous mutations (the same target site in both homologous chromosomes presented the same mutations) (Figure 3A). The 44 mutants were also analyzed with the Hi-TOM platform by preparing PCR samples according to the instruction of the platform (Supplementary Table 2). Results by using the two different methods confirmed that all the 18 albino plants were *DXS1* mutants, of which four plants (lines 2–43, 2–48, 3–16, and 3–42) were homozygous mutants, four plants (lines 2–44, 2–49, 3–43, and 3–45) were biallelic mutants, and 10 plants (lines 1–41, 2–12, 2–15, 2–45, 2–46, 2–47, 3–15, 3–41, 3–44, and 3–46) mutated at both sites of targets 1 and 2 (Figure 3B and Supplementary Table 2). Some of the 10 albino plants (lines 1–41, 2–12, 2–15, 2–45, 2–47, 3–15, 3–41, 3–44, and 3–46) with mutations at both of the target sites showed very complicated mutations, with more than one mutation at one target site (Figure 3B and Supplementary Table 2). These plants were chimeric mutants. The ratio of mutation was very different among the green SE plants from the three transgenic events, arranging from 7.9 to 28% (Table 3). Most of the green mutants were heterozygous mutants, but two SE plants showed two different mutated alleles at one target site (Supplementary Table 2 and Supplementary Figure 2), indicating that they were chimeric mutants. Genome-editing frequencies at target sites 1 and 2 were very different. Among the 44 mutants, 26 mutants showed genome editing at target site 1, 8 mutants showed mutations at target site 2, and 10 mutants showed mutations at both target sites 1 and 2, indicating higher genome-editing efficiency at target site 1 than at target site 2 (Supplementary Table 2). As 22.7% of the mutants showed genome editing at both target sites, the CRISPR/Cas9 toolbox developed in this research is useful for multi-sites genome editing. On the analysis, the mutations included 1-bp insertion, and deletions varying from 1 to 15 bp, and most of the mutations were about 3-bp upstream of the PAM site (Figure 3B and Supplementary Figure 2).

**Table 3:** The ratio of *DXS1* mutants in the green SE plants after genome editing.

**Transgenic events**	**Number of green plants for analysis**	**Number of mutant plants**	**Percentage of mutant plants (%)**
R1	63	5	7.9
R2	50	14	28.0
R3	52	7	13.5

## Discussion

**Figure 4:**
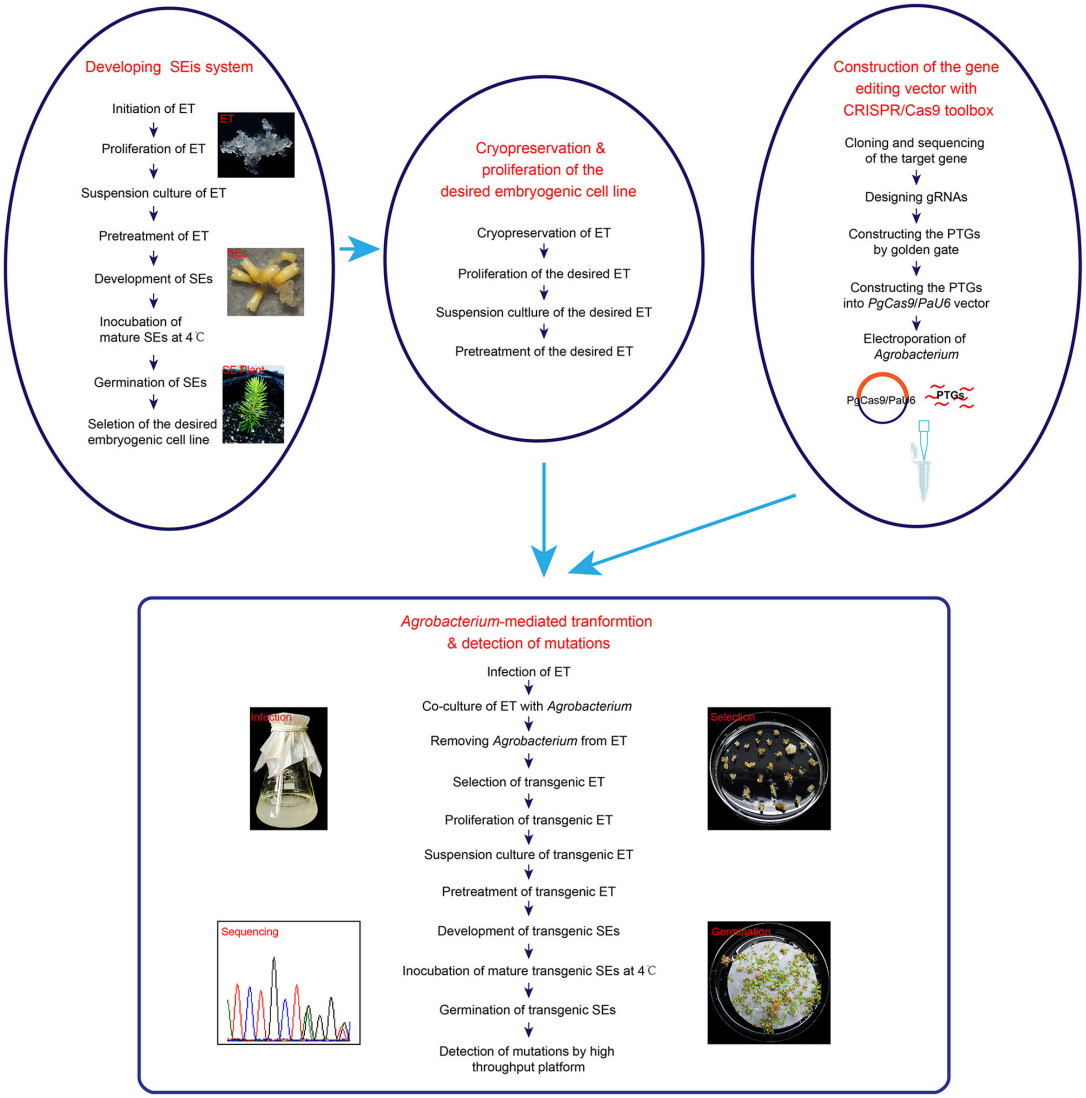
The process of somatic embryogenesis (SEis) and genome editing in *Picea glauca*. The procedure of developing a SEis system and genome editing with the desired embryogenic cell line is shown in this diagram. ET, SEis, SEs, and PTGs stand for embryogenic tissue, somatic embryogenesis, somatic embryos, and polycistronic tRNA-gRNAs, respectively.

In this report, the *DXS1* gene was edited successfully, which is the first successful stable genome editing and plant regeneration by using the CRISPR/Cas9 system in white spruce. Our success in genome editing in white spruce depends mainly on two aspects as summarized in Figure 4. First, a stable transformation and regeneration system is needed for developing genome-editing methods with CRISPR/Cas9 technique. Most coniferous species are difficult to regenerate and recalcitrant to genetic transformation (Sarmast, 2016), which severely hampers the application of genome-editing technologies in these plants. White spruce is a very important economic tree species in North America and a model species for research. White spruce can be regenerated *via* SEis and be genetically transformed by gene bombardment (Bommineni et al., 1993) or *Agrobacterium*-mediated transformation (Klimaszewska et al., 2001; Le et al., 2001). Although genetic transformation in white spruce was reported more than 20 years ago, no further reports about genetic transformation and genome editing are available with this species. In order to achieve genome editing in white spruce, the establishment of an *Agrobacterium*-mediated system of stable transformation frequency was important as the first step.

Second, we developed a CRISPR/Cas9 system suitable for genome editing in conifers. Since the codon bias in conifers was very different from that in other plants and the *U6* promoter was species-specific (Wang P. et al., 2018; Ren C. et al., 2021), we analyzed the codon-optimized *SpCas9* gene from a widely used plant genome-editing binary vector pYLCRISPR/Cas9 (Ma et al., 2015) and the conserved sequence of spruce *U6* promoter. The CAI of the *SpCas9* from pYLCRISPR/Cas9 in white spruce was only 0.67 (analyzed by GenScriptOptimumGene^TM^ tool), indicating very low expression in white spruce. So, *SpCas9* was codon-optimized according to codon bias in white spruce; after which, the CAI of itin white spruce was improved to 0.93. When the spruce *U6* promoter was analyzed, it showed different conserved USE, compared with the *AtU6* promoter. Therefore, the spruce *U6* promoter was cloned, and its function was verified in embryogenic tissues of both white spruce and larch. Both the codon-optimized *SpCas9* and the spruce *U6* promoter were used to construct the CRISPR/Cas9 toolbox. The results confirmed that the toolbox we designed, i.e., *PgCas9/PaU6*, was efficient in white spruce. It would also be efficient in *Larix* according to the function of the *PaU6* promoter in this genus. Additionally, the recently published reports on genome editing in conifers (Nanasato et al., 2021; Poovaiah et al., 2021) have indicated that some improvements on the CRISPR/Cas9 toolbox are worthy to be carried out in further studies, such as using the *Ubi* promoter to drive the expression of *Cas9* and/or testing some other *U6* promoters.

The polycistronic tRNA-gRNA (PTG) structure (Xie et al., 2015) had been widely used in multi-sites genome editing. In this research, two gRNAs were constructed into the PTG structure, and the result showed that proximately 22.7% of the mutations happened at both of the target sites 1 and 2. Thus, the PTG structure could be used for multi-sites genome editing at least in white spruce.

The mutation induced by CRISPR/Cas9 in this research included 1 bp insertions, and 1–15 bp deletions near the PAM sites, which was similar to the CRISPR/Cas9-mediated mutations in angiosperms. This fact indicates that the functions of the CRISPR/Cas9 system on conifers followed the rule demonstrated by the species that were previously studied. Hence, the application of many Cas9 variants, such as eSpCas9 (Slaymaker et al., 2016), SpCas9-HF1 (Kleinstiver et al., 2016), SpCas9-NGv1 (Endo et al., 2019), SpRY (Walton et al., 2020) in coniferous species, was promising. In addition, some precise genome-editing techniques, such as base editing (Komor et al., 2016; Gaudelli et al., 2017) and primer editing (Anzalone et al., 2019), which rely on the function of CRISPR/Cas9, were also worthy to be tested in conifers. Indeed, the SpRY was confirmed to be efficient with larch protoplasts (Ren Q. et al., 2021). With well-established systems of stable transformation and plant regeneration, techniques based on CRISPR/Cas9 could be used commonly in coniferous species in the near future. Interestingly, there were no large deletions between the two target sites in this research, whereas large deletions between two target sites were reported both in *Pinus radiata* (Poovaiah et al., 2021) and *Cryptomeria japonica* (Nanasato et al., 2021). Since only one gene was edited in this research, we could not make a conclusion on large deletions at other sites of the genome.

It was noted that the mutants developed from the same transgenic events showed a wide variety of mutations in this research. The chimeric phenomenon also existed in the same SE plants as they showed more than two kinds of variations at one target site. In citrus, the cause of chimeric mutations was attributed to the active divisions of the cells competent for transformation (Peng et al., 2017). In animals, the mosaicism when using the CRISPR/Cas9 system in embryos was predicted to be induced by the translational delay of Cas9 during cell divisions (Mehravar et al., 2019). Another possible cause that resulted in the chimeric mutation might be the culture temperature applied in this research, which was significantly lower than the most suitable temperature, about 37°C, for CRISPR/Cas9 system (Xiang et al., 2017). A high frequency of chimerism during genome editing in conifers was also reported by Poovaiah et al. (2021) and Nanasato et al. (2021). Poovaiah et al. (2021) thought the delayed Cas9 cleavage in the proliferating embryogenic tissue was the reason for chimerism, while Nanasato et al. (2021) thought the ongoing function of CRISPR/Cas9 led to chimerism. The exact reason for chimerism in this research was unclear, although the active division of embryogenic cells and the unfavorable temperature for Cas9 activity might be the cause. The major disadvantage of chimerism was that more plants were needed for the detection and selection to obtain the desired mutants. On the other side, chimerism helped us to deal with the embarrassment that insufficient transgenic events were obtained in some recalcitrant conifers since more than one kind of mutation could be found in the same transgenic events. In addition, the disadvantage could be overcome with a high-throughput detection platform, such as Hi-TOM (Liu et al., 2019). Although there was chimerism in this research, four homozygous mutants and four biallelic mutants were confirmed by the methods of both Sanger sequencing and Hi-TOM, which indicated that gene knockout mutants could develop at T_0_ generation. As conifers were perennial and cross-pollinated, developing homozygous mutants at T_0_ generation was especially important.

## Conclusion

We successfully knocked out the *DXS1* gene of white spruce by using a CRISPR/Cas9 toolbox designed for conifers in this study. Both homozygous mutants and biallelic mutants were obtained *via* SEis at T_0_ generation. We confirmed that the application of CRISPR/Cas9 was feasible in *Picea glauca*. The high frequency of chimerism, which was reported in *Pinus radiata* and *Cryptomeria japonica* (Nanasato et al., 2021; Poovaiah et al., 2021) after genome editing, was also found in this research, although a completely different genome-editing vector and another plant species were used during this research. According to the current study, we also illustrated the entire process of regenerating the genome-edited plants by application of CRISPR/Cas9 *via* SEis (Figure 4). Further research on the CRISPR/Cas9 system based on SEis could be focused on how to uncover and/or control chimerism during genome editing in conifers.

## Data Availability Statement

The original contributions presented in the study are included in the article/Supplementary Materials, further inquiries can be directed to the corresponding authors.

## Author Contributions

YC, LK, JZhang, and JZhao designed the experiments and revised the manuscript. YC, JZhao, YG, and RZ performed the experiments. YC analyzed the data and prepared the manuscript draft. All authors have read and approved the final manuscript.

## Funding

This paper was supported by the National Key R&D Program of China (nos. 2017YDF0600404-1 and 2017YDF0600501), the project fund (Somatic embryogenesis and high-efficient propagation technology in trees) provided by Beijing Advanced Innovation Center for Tree Breeding by Molecular Design, National Natural Science Foundation of China (no. 31901289), Key R&D Program of Heibei Province, China (no. 20326333D), and Major Science and Technology Special Project of Xuchang, Henan province, China (no. 20170112006).

## Conflict of Interest

The authors declare that the research was conducted in the absence of any commercial or financial relationships that could be construed as a potential conflict of interest.

## Publisher's Note

All claims expressed in this article are solely those of the authors and do not necessarily represent those of their affiliated organizations, or those of the publisher, the editors and the reviewers. Any product that may be evaluated in this article, or claim that may be made by its manufacturer, is not guaranteed or endorsed by the publisher.
