# Supplementary material for: Efficient Multi-Sites Genome Editing and Plant Regeneration via Somatic Embryogenesis in Picea glauca
Source: Front Plant Sci. 2021 Oct 14;12:751891. doi: 10.3389/fpls.2021.751891 (PMC8551722; doi:10.3389/fpls.2021.751891)
Supplement: Supplementary file 1 [file Data_Sheet_1.pdf]

Supplementary materials

1. Supplementary Figures

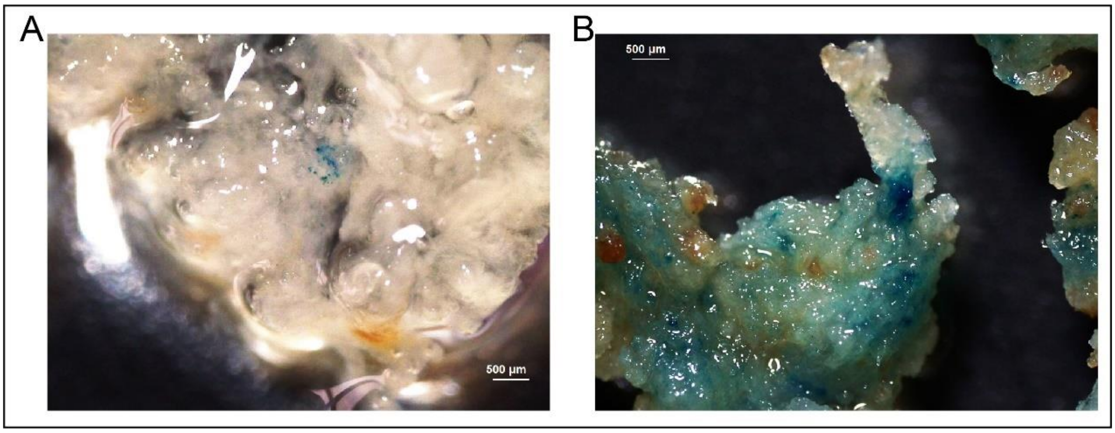

Supplementary Figure 1. The expression of *PaU6:GUS* in the embryogenic tissue of white spruce and Prince Rupprecht's larch.

Expression of *PaU6:GUS* in embryogenic tissue in white spruce and Prince Rupprecht's larch was shown in (A) and (B) respectively.

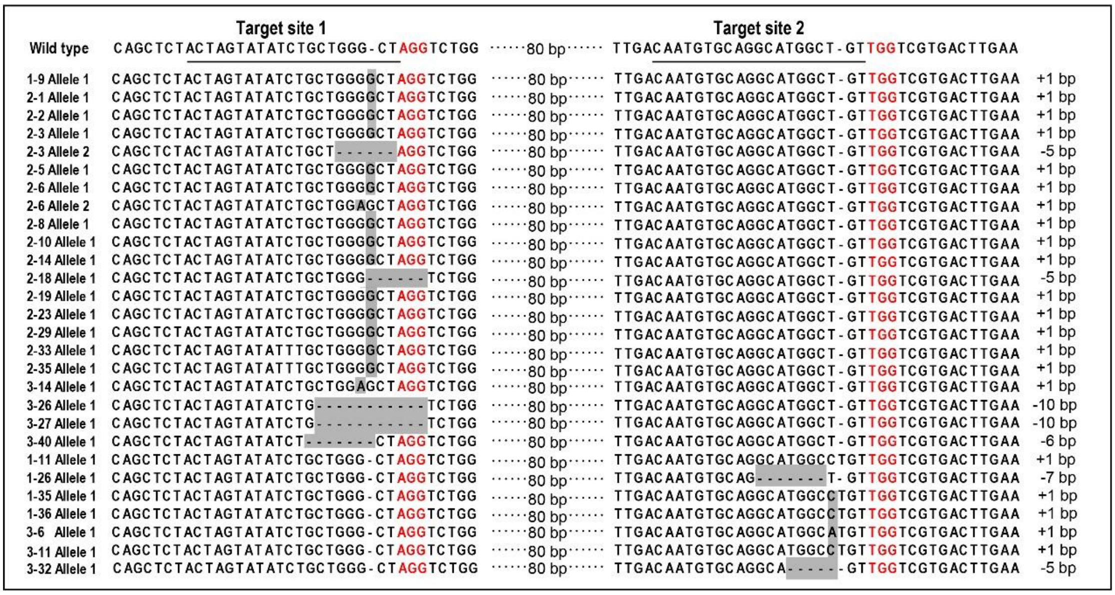

Supplementary Figure 2. The sequences at the target site in green mutant SE plants

The sequences at the target site 1 and the target site 2 in the wild type WSP3 somatic plants were underlined. PAM sites were indicated with red color. Individual transgenic somatic plant line was indicated by the plant line number on the left side of the sequence. Nucleotide insertion and deletion were indicated by +n bp and -n bp respectively on the right side of the sequence.

2. Supplementary Tables

Supplementary Table 1. Sequence information.

>*Pa U6* promoter (the USE element was indicated with yellow background)<br>CCGGGTTCAAGTCCCGGCAACGGAAATTAAATTGTTTTATAGGATTTTAAACAACATTGCAGAGATCTTCC<br>GGGTTTTCAATCGAGAAATCTGAAATTCTCTAAACAGAAAAGAGTAAACACAGGTGCGTCTGCCGGGAG<br>TCGAACCCGGGTCTATTGCTTGGAAGGCAATTATCCTAACCGTTGGACTACAAACGCCTAGATGTAAGAG<br>GTTAACAAGAGGGGCATACCAGGCTATTAATCTAACAGTTGGACATGCAAACCACAAAATGTGAGAGCG<br>GATACATGCTTTTCGTTGATAATATTACTACAACTGGACTATTACTTTAACAGTTGGACTACAACTGCTCTC<br>GGTGTAACCGCTGGACAATAAACGCTACTTGCAAACCACAAAATGTGAGAGCGTTTCGCTGATAATAATA<br>CTAAATTCGTTGCACTCCTCGAGTGTTAAAATTCAAACCCCAAATCTCACGCAAACGCAAATTCAAACAG<br>AAGAGATACAGATATACACGGAGGGGACATCCGTAACGCGAAATAGATATACATTTTTCGTATAACACTTG<br>CAGTTTATTCCCACATGCGTAAAGAAAAATAGATATAAACCTTTCATATACGGAGATGATAGCTTAAGTTGT<br>TGTCTCTTCG
> WSP3 *DXS1* (sequence which was amplified with the primer DXS-4F and DXS-5R)<br>AGACAGACAAATGGCCTCTCAGGCTTTACAAAACGTTCAGAGAGTGAATATGACTGCTTTGGTGCCGGT<br>CACAGCTCTACTAGTATATCTGCTGGGCTAGGTCTGGTTCTGGGCATCTTCAGATATTTGAATGTTATGAGT<br>TTTCCTACAGGAATTGGTATAGAAGGGGACTTTAACTCTGACCATTTGACAATGTGCAGGCATGGCTGTT<br>GGTCGTGACTTGAAAGGGAGAAACAATCATGTCATTAGTGTCATTGGAGATGGAGCCATGACAGCTGGG<br>CAAGCCTTTGAAGCTATGAACAATGCTGGATATTTGGATTCCAACATGATAGTTATCCTGAATGACAACAA<br>ACAAGTTTCTCTGCCAACGGCAAATCTTGATGGGCCTATGCCACCAGTGGGTGCTCTCAGCAGTGCACTG<br>AAGGTAAGCTG

Supplementary Table 2. The variation type at the target site detected by Hi-TOM platform

SE plants	Allele	Reads number	Ratio	Target site 1 variation type	Target site 2 variation type
1-9	1	1406	48.25%	1I	WT
	2	1258	43.17%	WT	WT
1-11	1	1392	46.79%	WT	WT
	2	1310	44.03%	WT	1I
1-26	1	2217	74.17%	WT	WT
	2	416	13.92%	WT	7D
1-35	1	1486	50.27%	WT	WT
	2	1470	49.73%	WT	1I
1-36	1	1296	43.74%	WT	WT
	2	1267	42.76%	WT	1I
1-41	1	1221	41.74%	15D	WT
	2	901	30.80%	WT	1I
	3	417	14.26%	15D	1I
	4	386	13.20%	WT	WT
2-1	1	1753	60.08%	WT	WT
	2	870	29.81%	1I	WT

2-2	1	1615	54.89%	WT	WT
	2	988	33.58%	1I	WT
2-12	1	1040	35.17%	1I	WT
	2	1030	34.83%	WT	8D
	3	446	15.08%	WT	WT
	4	369	12.48%	1I	8D
2-5	1	1346	45.32%	1I	WT
	2	1326	44.65%	WT	WT
2-3	1	1368	46.23%	WT	WT
	2	800	27.04%	5D	WT
	3	656	22.17%	1I	WT
2-6	1	1692	57.67%	WT	WT
	2	797	27.16%	1I	WT
	3	348	11.86%	1I	WT
2-8	1	1615	54.75%	WT	WT
	2	1157	39.22%	1I	WT
2-10	1	1939	64.87%	WT	WT
	2	380	12.71%	1I	WT
2-14	1	1352	46.24%	WT	WT
	2	1293	44.22%	1I	WT
2-15	1	1100	37.47%	5D	WT
	2	1078	36.72%	1I	WT
	3	495	16.86%	5D	5D
2-18	1	1470	49.56%	WT	WT
	2	1115	37.59%	5D	WT
2-19	1	1354	46.56%	WT	WT
	2	1311	45.08%	1I	WT
2-23	1	1518	52.15%	WT	WT
	2	1133	38.92%	1I	WT
2-29	1	1374	47.23%	1I	WT
	2	1374	47.23%	WT	WT
2-33	1	1524	52.23%	1I	WT
	2	1123	38.49%	WT	WT
2-35	1	2062	69.76%	1I	WT
	2	700	23.68%	WT	WT
2-43	1	2413	80.84%	1I	WT
2-44	1	2056	69.58%	1D	WT
	2	609	20.61%	1I	WT
2-45	1	1039	35.47%	WT	1I
	2	908	31.00%	7D	WT
	3	456	15.57%	WT	WT
	4	299	10.21%	7D	1I
2-46	1	1759	59.51%	1I	WT
	2	655	22.16%	1I	4D

2-47	1	1200	40.42%	1D	WT
	2	1112	37.45%	1I	WT
	3	409	13.78%	1I	1I
2-48	1	2481	84.47%	1D	WT
2-49	1	1767	60.43%	1D	WT
	2	1051	35.94%	1I	WT
3-6	1	1870	63.45%	WT	WT
	2	661	22.43%	WT	1I
3-11	1	2308	79.12%	WT	WT
	2	227	7.78%	WT	1I
3-14	1	1357	45.74%	WT	WT
	2	1323	44.59%	1I	WT
3-15	1	920	30.81%	WT	WT
	2	582	19.49%	5D	1I
	3	323	10.82%	1D	WT
	4	298	9.98%	1D,7D	5D
3-16	1	2645	90.12%	1D	WT
3-26	1	1144	38.32%	10D	WT
	2	1023	34.27%	WT	WT
3-27	1	1610	54.30%	WT	WT
	2	985	33.22%	10D	WT
3-32	1	1539	52.29%	WT	WT
	2	1192	40.50%	WT	5D
3-40	1	1189	39.81%	WT	WT
	2	717	24.00%	6D	WT
3-41	1	1168	39.12%	5D	WT
	2	832	27.86%	WT	5D
	3	370	12.39%	WT	WT
	4	339	11.35%	5D	5D
3-42	1	2678	89.57%	1D	WT
3-43	1	1313	44.63%	6D	WT
	2	1114	37.87%	1I	WT
3-44	1	941	32.28%	WT	1I
	2	607	20.82%	1D	2D
	3	593	20.34%	1D	1I
	4	354	12.14%	1D	1I
3-45	1	1652	56.08%	WT	1D
	2	1217	41.31%	WT	1I
3-46	1	1208	40.52%	2D	WT
	2	468	15.70%	2D	1D
	3	359	12.04%	WT	1D

The PCR products which were prepared according to the instruction of Hi-TOM platform were sequenced by next-generation sequencing. More than 1000 reads were analyzed. The mutations with a ratio > 10% were shown in this table, except 3-11, which showed a clear double chromatogram near the PAM site but a low ratio of mutations.

3. Supplementary methods

Supplementary Method 1. Construction of the genome editing vector targeting the *DXS1* with *PgCas9/PaU6* toolbox.

The method of assembling polycistronic tRNA-gRNA (PTG) was based on Xie et al., (2015) with some modifications. The sequence of the amplification template of gRNA-tRNA was the same to that described by Xie et al., (2015), except that gRNA-tRNA was cloned into the plasmid PUC57. To assemble gRNA1 and gRNA2 from *DXS1*, the specific primers were designed and shown in the following table.

gRNA1-F:	TAGGTCTCAATCTGCTGGGCTGTTTTAGAGCTAGAAATAG
gRNA1-R:	CGGGTCTCCAGATATACTAGTTGCACCAGCCGGGAATC
gRNA2-F:	TAGGTCTCAAGGCATGGCTGTGTTTTAGAGCTAGAAATAG
gRNA2-R:	CGGGTCTCCGCCTGCACATTGTGCACCAGCCGGGAATC
U-F:	CGGGTCTCATTCGAACAAAGCACCAGTGGTC
U-R:	TAGGTCTCCAAACAAAAAAAAAAGCACCGACTCGGTGCCAC

Three PCR amplifications were then performed respectively with the primer pairs U-F/ gRNA1-R，gRNA1-F/ gRNA2-R and gRNA2-F/U-R in a PCR reaction system and a PCR progress shown below.

The first PCR reaction system:

PUC57（gRNA-tRNA） plasmid	0.1 ng
10×KOD buffer	2 μL
dNTPs (8 mM)	2 μL
$MgSO_4$ (25 mM)	0.8 μL
Forward primer (10 μM)	0.6 μL
Reverse primer (10 μM)	0.6 μL
KOD (1U/μL) KOD-401, TOYOBO	0.4 μL
ddH_2O	X μL
Total	20 μL

The first PCR reaction progress:

Temperature	time	cycles
95℃	3 min	1
95℃	10 sec	30
58℃	15 sec	
68℃	20 sec	
68℃	2 min	1
4℃	Hold	1

The three PCR products were purified with Universal DNA Purification Kit, (DP214, TIANGEN BIOTECH CO., LTD, Beijing, China) and ligated together with the first golden gate assembly with the following reaction system.

Each of the three PCR products	20 ng
10×T_4 DNA ligase Buffer	2 μL
BsaI (10 U/μL) (R0535V, NEB)	0.5 μL
T_4DNA ligase (400 U/μL) (M0202V, NEB)	0.1 μL
ddH_2O	X μL
Total volume	20 μL

The first golden gate assembly reaction was performed in a thermal cycler by incubation at 37℃, 5 min and 20℃, 5 min for 15 cycles. The golden gate reaction product was then diluted with 180 μL ddH_2O, and amplified in the 50 μL PCR reaction with the primer U-2F (5'- CGGGTCTCATTCG AACAA-3') and U-2R (5'-TAGGTCTCCAAACAAAAAAAAAAGCACCG-3'). The second PCR reaction system and progress were shown below.

The second PCR reaction system:

Ligation product (1:10 dilution)	3 μL
10×KOD buffer	3 μL
dNTPs (8 mM)	3 μL
$MgSO_4$ (25 mM)	1.2 μL
U-2F (10 μM)	0.9 μL
U-2R (10 μM)	0.9 μL
KOD (1U/μL) (KOD-401, TOYOBO)	0.6 μL
H2O	X μL
Total	30 μL

The second PCR progress:

temperature	time	cycles
95℃	2 min	1
95℃	10 sec	30
58℃	15 sec	
68℃	40 sec	
68℃	2 min	1
4℃	Hold	1

The PCR product was purified with the Universal DNA Purification Kit, (DP214, TIANGEN BIOTECH CO., LTD, Beijing, China), and ligated together by the second golden gate assembly with the following reaction system:

PgCas9/PaU6a plasmid	100 ng
PCR product	50 ng
10×*Bsa*Ibuffer	1.5 μL
ATP (10 mM) (P0756S, NEB)	1.5 μL
*Bsa*I (10 U/μL) (R0535V, NEB)	1 μL
T_4DNA ligase (400 U/μL)(M0202V, NEB)	0.1 μL
ddH_2O	X μL
Total volume	15 μL

The second golden gate reactions were performed in a thermal cycler by incubation at 37℃, 2 min;10℃, 3 min, 20℃, 5 min for 15 cycles, at last hold at 37℃ for 2 min.

The ligation product were then transferred into *E.coli* TOP10, and the positive clone were selected by PCR amplification. The PCR positive clones were further verified by digesting the positive clones with the recognize restriction enzyme *Hind*III and *Bam*HI, *Hind*III and *Eco*RI. The true positive clone would produce a fragment with a size of 1.3 kb and 6.6 kb in the two digesting reactions respectively. The final positive plasmid was transformed into the *Agrobacterium* strain *EHA*105.

References:

Xie, K., Minkenberg, B., Yang, Y. (2015). Boosting CRISPR/Cas9 multiplex editing capability with the endogenous

tRNA-processing system. PNAS 112, 3570-3575.
